# Correspondence

**Published:** 1904-10

**Authors:** H. K. Leake

**Affiliations:** Dallas, Texas


### Correspondence.

DALLAS, TEXAS, September 18, 1904.

*Dear Dr. Daniel:*

I have returned from New York and Philadelphia, where I spent several weeks renewing my old surgical acquaintances. Since I was there two years ago, I note little, if any, change in the technique and in the after-treatment of surgical cases. The best operators seemed to be fixed in their methods, their mortality being now so low that, apparently, there is little room for improvement in the essential features of operation cases. Conspicuously this is true of my friends Price and Deaver, of Philadelphia, who stand unsurpassed as successful operators in this country. If there be any change in the methods of these men it is in the direction of accurate diagnosis and simplicity in all the details of an operation.

It appears that the well-advertised assertion of a great English surgeon twenty years ago that "surgical methods have now reached perfection" is about to be realized, although much remains to be done concerning diagnosis and pathology. I was struck with the remark of a prominent operator that "the question now is not how, but when, to operate," and, of course, this question can be answered only by close and intelligent study of all the diagnostic and pathologic aspects of every case presented to the surgeon. Speaking broadly then, widespread revision of all our knowledge affecting surgical cases is now going on throughout the country, and this revolution is at the supreme dictation of that *mens conscia recti* that should control the surgeon as well as every other individual in all the relations of life. Consequently the "operative furor" is being tempered by a wiser and more conservative judgment than formerly, and this progressive wave will reach Texas in a very short time.

While in New York I was shocked by the death of Dr. Pryor, who had reached such eminence in his special line of work. His death spread a pall over the entire New York medical community. It it said that he was a man full of noble impulses that had endeared him even to his rivals. He died of leukemia that was diagnosticated only a few days before his death, the microscopical examination of the blood having been made by Dr. Jeffries of the Polyclinic Medical College. Before his death, Dr. Pryor declared that he regretted that he must go before his work was finished. He was only 42 years of age, and started his career as an assistant of Dr. Harry Marion Sims, who survives him.

I am pleased to inform you of the growing popularity of your journal, and of the satisfaction of your many friends that you were elected President of the State Medical Association—an honor long since deserved.

Sincerely yours,

H. K. Leake, M. D.

